# The race between 4-1BB- and CD28-based CD19 CAR-T products in the therapy of B-cell malignancies

**DOI:** 10.1016/j.bbcan.2025.189519

**Published:** 2026-02

**Authors:** Marta Krawczyk, Magdalena Drużyńska, Emilia Bednarska, Magdalena Winiarska

**Affiliations:** a Department of Immunology, Mossakowski Medical Research Institute, Polish Academy of Sciences, Warsaw, Poland; b Doctoral School of Translational Medicine, Mossakowski Medical Research Institute, Polish Academy of Sciences, Centre of Postgraduate Medical Education, Warsaw, Poland; c Immunooncology Students' Science Association, Medical University of Warsaw, Warsaw, Poland

**Keywords:** CD19, CAR-T, Costimulatory domain, Immunotherapy, Resistance

## Abstract

Chimeric antigen receptor T-cell (CAR-T) therapy targeting CD19 has revolutionized the treatment of B-cell malignancies. One of the critical factors influencing CAR-T efficacy and durability is the costimulatory domain, with 4-1BB and CD28 emerging as the two dominant signaling platforms. While CD28-based CAR-T cells exhibit strong initial potency and rapid expansion, 4-1BB-based CAR-T cells demonstrate greater persistence and long-term efficacy. However, resistance to CAR-T therapy remains a significant challenge. Tumor cells develop a variety of mechanisms to evade immune surveillance, including CD19 antigen escape due to epigenetic factors or genetic aberrations of the *CD19* gene. This review article summarizes the mechanistic differences between both costimulatory domains, their impact on clinical outcomes, and how they might influence resistance occurrence. By dissecting the battle of potency and the race of persistence, we provide insights into the evolving landscape of CAR-T therapy for B-cell malignancies.

## Highlights

• CD19 loss is the dominant resistance mechanism after CD19-directed CAR-T therapy.
• The costimulatory domain is one of the factors influencing the CAR-T cells' response.
• CD28 CARs act more potently, while 4-1BB CARs persist longer.
• CD28-based CAR-T therapy is less prone to select CD19^low^ tumor cells than 4-1BB-based.

## Graphical abstract

**Figure:**
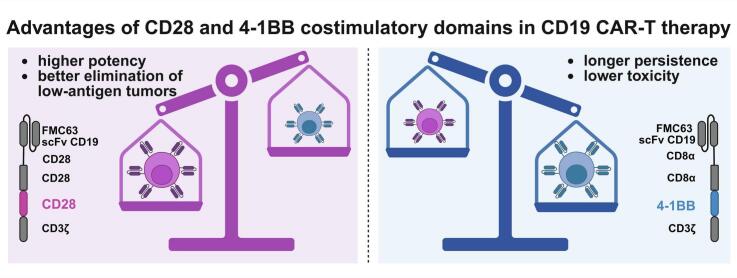

## 1 Introduction

Recent years have seen remarkable advancements in hemato-oncology, particularly following the development of cancer immunotherapies. Among these, adoptive cell therapy using chimeric antigen receptor T-cell (CAR-T) targeting CD19 has been a major breakthrough. Since 2017, four anti-CD19 CAR-T products based on FMC63 single-chain variable fragment (scFv) and one low-affinity CAT (CAT13.1E10)-based scFv have been approved for treating relapsed/refractory (r/r) high-grade B-cell malignancies. These therapies, incorporating either 4-1BB or CD28 costimulatory domains, achieved up to 74 % complete remission rates in the initial clinical trials [[1], [2], [3], [4], [5]].

Despite their phenomenal results, CD19 CAR-T therapies face several challenges. A significant subset of high-grade lymphoma and B-cell acute lymphoblastic leukemia (B-ALL) patients exhibit primary or acquired resistance, with mechanisms including antigen escape of tumor cells, CAR-T cell exhaustion, and the immunosuppressive function of tumor microenvironment. Up to now, the most commonly reported cause of resistance to CD19 CAR-T therapy has been CD19 antigen loss [6,7]. This mainly occurs through *CD19* genetic alterations disrupting the FMC63 epitope that help tumor cells escape CAR-T recognition [8,9]. Moreover, clinical reports show that in nearly 17 % of B-ALL patients CD19-negative cells are detected before treatment, further contributing to the relapse [10].

Despite the extensive research, the impact of costimulatory domains on resistance mechanisms remains poorly understood. 4-1BB and CD28 CAR costimulatory domains are critical for CD19 CAR-T efficacy and durability. It is widely reported that CAR-T products with 4-1BB and CD28 costimulatory domains differ in function. While CD19-4-1BB CAR-T cells persist longer and proliferate faster, CD19-CD28 CAR-T cells act more potently and rapidly [[11], [12], [13]].

Herein, we provide insight into the mechanistic differences between 4-1BB- and CD28-based CAR-T cells and how these variabilities influence the clinical outcome of the therapy and shape the development of the resistance process.

## 2 Available CD19 CAR-T therapies

CAR is a synthetic molecule with a modular structure that includes: 1) extracellular antibody-derived domain – scFv, responsible for antigen recognition, 2) hinge, 3) transmembrane helix (TM), 4) one or two costimulatory domains (e.g. CD28, 4-1BB, ICOS, OX40), and 5) intracellular activating domain derived from T cell receptor (CD3ζ chain) [14,15].

**Fig. 1:**
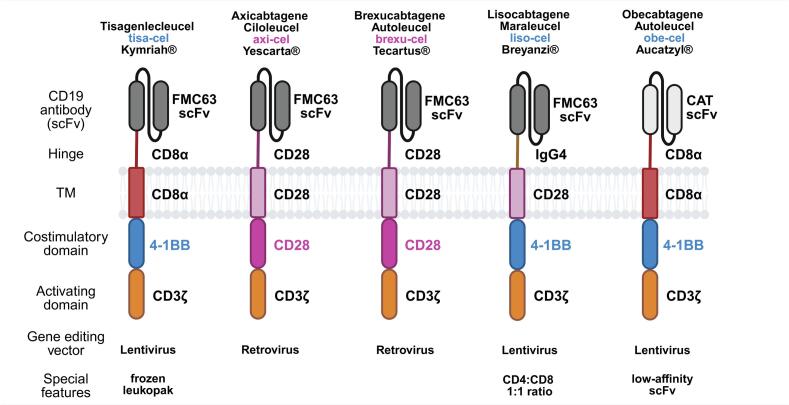
The structures and features of CAR utilized in the CD19-targeting clinical products. 4-1BB-based products, including tisa-cel, liso-cel and obe-cel, and CD28-based products – axi-cel and brexu-cel.

Since 2017, five different anti-CD19 CAR-T products (four built with FMC63 scFv and one with low-affinity CAT scFv) have been approved by the Food and Drugs Administration (FDA) and European Medicines Agency (EMA). Kymriah (Tisagenlecleucel), Breyanzi (Lisocabtagene Maraleucel) and Aucatzyl (Obecabtagene Autoleucel) utilize the 4-1BB costimulatory domain, while Yescarta (Axicabtagene Ciloleucel) and Tecartus (Brexucabtagene Autoleucel) are based on the CD28 costimulatory domain (Fig. 1).

The initial clinical trials of all five therapeutics revealed phenomenal results, which are summarized in Table 1. and below.

**Table 1:** Clinically approved CD19 CAR-T products.

Generic/Brand Name	Manufacturer	Costimulatory Domain	Clinical Indications	Approval Date (FDA/EMA)	CR	Ref.
Tisagenlecleucel (tisa-cel) Kymriah®	Novartis	4-1BB	r/r pediatric and YA B-ALL	August 2017/August 2018	60 %	[16]
			r/r DLBCL	May 2018/August 2018	40 %	[17]
			r/r FL	May 2022/April 2022	69 %	[18]
Axicabtagene Ciloleucel (axi-cel) Yescarta®	Kite Pharma Inc.	CD28	r/r adult DLBCL	October 2017/August 2018	54 %	[19]
			r/r adult PMBCL			
			r/r adult FL and MZL	March 2021/April 2022	74 %	[20]
Brexucabtagene Autoleucel (brexu-cel) Tecartus®	Kite Pharma Inc.	CD28	r/r adult MCL	July 2020/December 2020	67 %	[21]
			r/r adult B-ALL	October 2021/July 2022	53 %	[22]
Lisocabtagene Maraleucel (liso-cel) Breyanzi®	Juno Therapeutics, Inc., a Bristol-Myers Squibb	4-1BB	r/r DLBCL, r/r PMBCL, r/r FL3B	February 2021/March 2023	53 %	[23]
			r/r B-CLL/B-SLL	May 2024/NYR	20 %	[24]
			r/r FL	May 2024/March 2025	73 %	[25]
			r/r MCL	May 2024/NYR	68 %	[26]
Obecabtagene Autoleucel (obe-cel) Aucatzyl®	Autolus GmbH	4-1BB	r/r adult B-ALL	November 2024/pending	55 %	[27]

CR – complete response; YA – young adults; B-ALL – B-cell acute lymphoblastic leukemia; DLBCL – diffuse large B-cell lymphoma, FL- follicular lymphoma; PMBCL – primary mediastinal large B-cell lymphoma; MZL – marginal zone lymphoma; MCL – mantle cell lymphoma; FL3B – grade 3B FL; B-CLL – B-cell chronic lymphocytic leukemia; B-SLL – B-cell small lymphocytic lymphoma; NYR – not yet registered

### 2.1 4-1BB-based CAR-T products

Tisa-cel was the first CAR-T product ever approved [1], initially receiving FDA approval in August 2017 for the treatment of r/r B-ALL in patients up to 25 years old. This decision was based on results from the ELIANA trial, where an objective response rate (ORR) of 81 % and a complete response (CR) rate of 60 % were observed within three months after infusion [16]. Beyond B-ALL, it has also been approved for r/r diffuse large B-cell lymphoma (DLBCL) based on the JULIET trial, where an ORR and CR reached 52 % and 40 %, respectively [17]. Thereafter, tisa-cel received approval for the treatment of r/r follicular lymphoma (FL) following promising results from the ELARA trial, which reported an ORR of 86 % and a CR rate of 69 % [18] .

Liso-cel has also demonstrated considerable efficacy in the treatment of multiple B-cell malignancies [3]. It was first approved in r/r DLBCL, primary mediastinal large B-cell lymphoma (PMBCL), and grade 3B FL (FL3B), based on the TRANSCEND NHL 001 trial, in which the recorded ORR was 73 % and 53 % of patients achieved CR [23]. Then, the FDA expanded liso-cel's indications to include r/r chronic lymphocytic leukemia (CLL) and small lymphocytic lymphoma (SLL), following promising results of the TRANSCEND CLL study. In this trial, ORR reached 45 %, with CR observed in 20 % of patients, and a median duration of response of 35.3 months [24]. More recently, in 2024, liso-cel's approval was further expanded to r/r FL and r/r mantle cell lymphoma (MCL). The decision regarding r/r FL was based on exceptional results from the phase II TRANSCEND FL trial, where ORR reached 96 %, and CR was observed in 73 % of patients. Notably, 77 % of patients maintained a response 18 months post-infusion [25]. Approval for r/r MCL followed the phase I TRANSCEND NHL 001 trial, which demonstrated an ORR of 85 % and a CR rate of 68 % in primary analysis across MCL cohort [26].

Obe-cel is the most recently registered CD19-targeted CAR-T product, approved by the FDA in November 2024 for r/r adult B-ALL treatment following the phase Ib/II FELIX trial [27]. The study enrolled 153 patients divided into two cohorts – main 2A cohort (94 patients with morphologic disease) and 2B (patients with measurable residual disease). In cohort 2A overall remission (OR) occurred in 77 % patients and CR in 55 %. The observed cytotoxicity was low – only 2.4 % of patients developed grade 3 or higher cytokine release syndrome (CRS) [27]. This product, incorporating lower affinity CAT scFv in comparison to classically used FMC63 scFv, was reported as a fast off-rate CAR, which was firstly tested in phase I CARPALL (NCT02443831) and ALLCAR19 (NCT02935257) clinical trials. The reduced affinity was hypothesized to be advantageous in terms of shorter target-effector contact, reduced cytokine release and T cell exhaustion, all of which may enhance the CAR-T cell persistence [28,29].

### 2.2 CD28-based CAR-T products

Axi-cel was the first CAR-T therapy approved for the DLBCL and PMBCL treatment [2]. The FDA approval came in October 2017 as a result of the ZUMA-1 trial, which showed an ORR of 82 % and a CR rate of 54 % [19]. The median time to initial response was 1 month while the median duration of response was 8.1 months. Afterward, axi-cel also received approval for the treatment of FL and marginal zone lymphoma (MZL). In phase II of the ZUMA-5 study at the median follow-up time of 17.5 months, the ORR was 92 % and 74 % of patients achieved CR [20].

Brexu-cel was the first CAR-T therapy in the treatment of MCL, approved by the FDA in July 2020 [4]. Reports from the ZUMA-2 study showed that brexu-cel provides significant improvement for MCL patients while causing serious but generally reversible adverse effects. The study reported an ORR of 93 % while CR was achieved in 67 % of patients at 12.3 months of follow-up and the median time for an initial response was 1 month [21]. Subsequently, the approval of brexu-cel was extended for the treatment of B-ALL based on the ZUMA-3 clinical trial, where the ORR reached 69 % and 53 % of treated participants achieved CR [22].

## 3 Comparison of 4-1BB- and CD28-based CD19 CAR-T products based on clinical settings

### 3.1 Long-term outcome studies

The results of long-term (median follow-up of 24–61 months) outcome studies of 4-1BB-based CD19 CAR-T clinical products in lymphoma patients revealed 44–88 % ORR and 39–55 % CR rates and median progression-free survival (PFS) of 1.4–6.8 months [[30], [31], [32], [33]]. On the other hand, the long-term (median follow-up of 25–51 months) outcome studies of CD28-incorporating CD19 CAR-T products suggested superior efficacy, where lymphoma patients achieved 74–91 % ORR, 54–68 % CR rate and median event-free survival (EFS) was 5.7 to even 55 months [[34], [35], [36], [37]].

Nevertheless, the long-term (median follow-up 13–57.6 months) observation studies in leukemia patients revealed similar efficacy (69–85 % CR – 4-1BB vs. 62–86 % CR – CD28) and EFS (5.6–24 months – 4-1BB vs. 3.1–17 months – CD28) for both 4-1BB-based and CD28-based CD19 CAR-T clinical products [[38], [39], [40], [41], [42], [43], [44], [45], [46], [47]]. The above results suggest that CD28-based CAR-T therapy outperforms the 4-1BB-based one in terms of efficacy and time to progression in lymphoma patients, while both show similar efficacy in B-ALL patients.

### 3.2 Real-world comparisons of 4-1BB- and CD28-based products in lymphoma and leukemia treatment

In lymphoma patients, the first comparison analysis of CD28-based axi-cel vs. 4-1BB-based tisa-cel was published in 2019 supported by ZUMA-1 and JULIET clinical trials. This indirect analysis showed favored axi-cel in ORR (relative risk (RR) = 1.62) and CR (RR = 1.56). Moreover, with the 24-month follow-up period, in the group of axi-cel-treated patients, the mean survival was 6.3 months longer than in tisa-cel-treated patients. Axi-cel, however, had a higher rate of grade 1-2 CRS (RR = 2.02), remaining more toxic than tisa-cel [48].

Furthermore, French DESCAR-T retrospective studies published in 2022 enrolled the propensity score matching (PSM)-based comparison of axi-cel and tisa-cel in DLBCL treatment. This study, including 809 patients, reported axi-cel superior outcome based on significantly higher ORR (80 % vs. 66 %), CR (60 % vs. 42 %), overall survival (OS) (63.5 % vs. 48.8 %) and 1-year PFS (46.6 % vs. 33.2 %), but also higher toxicity than tisa-cel [49]. Similarly, the Italian multicentre prospective observational study, CART-SIE (2024), compared axi-cel and tisa-cel outcomes in 485 patients with r/r LBCL. The results revealed higher ORR (59.2 % vs. 47.6 %), complete response rate (CRR) (74.7 % vs. 61.1 %) and higher 1-year PFS (46.5 % vs. 34.1 %) after axi-cel treatment in contrast to tisa-cel treatment, but again axi-cel showed higher toxicity [50].

The systematic reviews and meta-analysis studies published in 2020 and 2024 established the superiority of axi-cel over tisa-cel in terms of response to treatment, survival after therapy, and time to relapse, but also confirmed higher toxicity of axi-cel in lymphoma patients [[51], [52], [53]].

In leukemia patients, the direct comparison studies are limited and unbalanced, because the 4-1BB tisa-cel product remains the most commonly used for B-ALL treatment. The meta-analysis studies published in 2021 and 2023 showed superior or similar efficacy of 4-1BB-based CAR-T products, but also higher relapse rates (0.36 vs. 0.28) compared to CD28-based CAR-T cells in leukemia patients. The analysis of toxicity did not provide clear conclusions as in one study the 4-1BB-based CAR-T therapy showed higher toxicity and, in another, it was CD28-based CAR-T [54,55]. Furthermore, the study published in 2024 which compared tisa-cel and brexu-cel, including 69 patients (50 tisa-cel and 19 brexu-cel), showed similar efficacy of both products, but suggests more durable response in the case of brexu-cel, revealing also its higher toxicity [56].

**Fig. 2:**
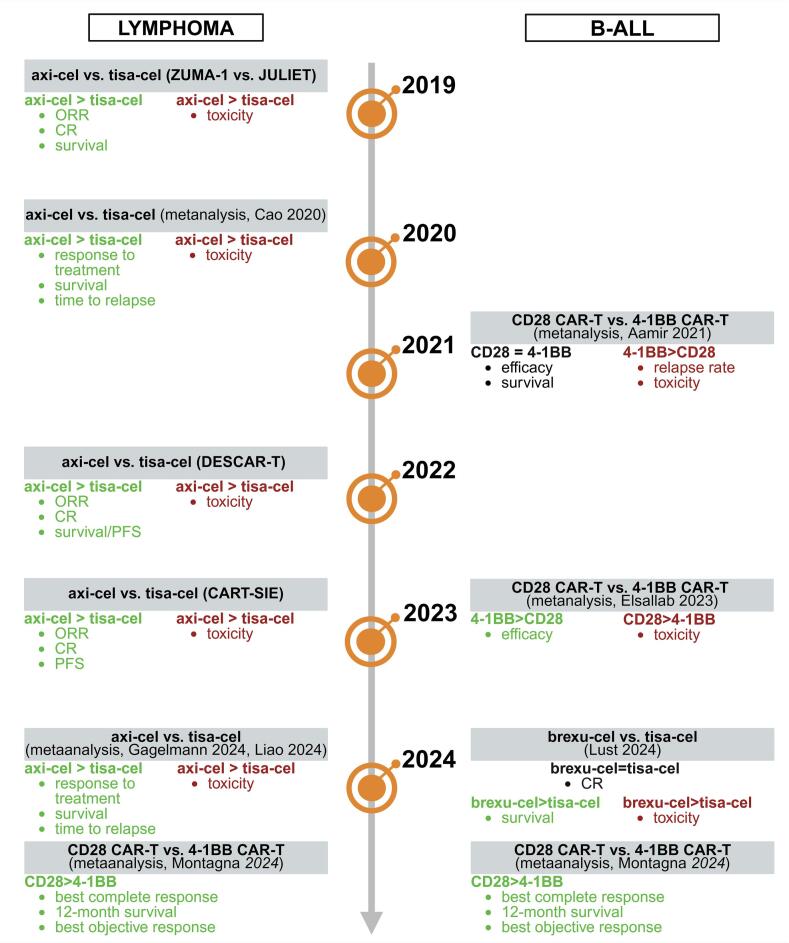
Summary of real-world comparisons between CD28-based and 4-1BB-based CD19 CAR-T therapies in B-ALL and lymphoma. The studies in lymphoma showed the superior efficacy of axi-cel over tisa-cel, revealing also the higher toxicity of axi-cel in all comparisons. The B-ALL data showed comparable efficacy of both, CD28- and 4-1BB-based products, but suggested that the toxicity of CD28-based products could be higher. The latest meta-analysis (Montagna 2024) comparing all available studies in both diseases revealed the superiority of CD28-based products over 4-1BB-based products.

Finally, Montagna et al. [57] in 2024 published the systematic review and meta-analysis, including all available clinical reports where CD19 CAR-T products were used for the treatment of both lymphoma (non-Hodgkin and Hodgkin lymphoma) or leukemia (ALL, CLL). The systematic review included 3493 patients across 56 studies, and the meta-analysis enrolled 3421 patients in 46 studies. Thus far, it was the most comprehensive comparison of CD19 CAR-T therapies clinical outcomes. The analysis was aimed, among other objectives, at assessing CAR structural domains and their clinical impact. The authors performed the analysis based on three therapy outcomes' parameters divided into primary outcome (BCR – best complete response) and secondary outcome (OS – 12-month overall survival and BOR – best objective response). The exact comparison of the most commonly used axi-cel and tisa-cel products showed better outcomes for axi-cel (62 % BCR, 68 % OS, 86 % BOR vs. 53 % BCR, 61 % OS, 70 % BOR for tisa-cel). Similarly, the general comparison of CD28 vs. 4-1BB costimulation revealed the superior outcome after CD28-based CD19 CAR-T therapy (60 % BCR, 66 % OS, 85 % BOR vs. 56 % BCR, 56 % OS, 71 % BOR for 4-1BB). Altogether, Montagna et al. showed that the presence of CD28 in the CAR structure, including hinge, TM, and costimulatory domains, improved response and 12-month survival after CD19 CAR-T therapy infusion [57], confirming the superior efficacy of CD28-based over 4-1BB-based CD19- therapy (Fig. 2).

## 4 Mechanism of action and preclinical comparison of 4-1BB- and CD28-based CD19 CAR-T cells

The reasons behind the reported differences in the efficacy and toxicity of 4-1BB- and CD28-based CD19 CAR-T clinical products, as reported both in vitro and in vivo*,* lie within the physiological role of these co-receptors in natural T cells response. A better understanding of their biological function might help to explain the observed diverse consequences in the context of CD19-4-1BB and CD19-CD28 CAR-T cells.

### 4.1 Costimulatory 4-1BB and CD28 receptors in T cell activation

The natural T cell response is dependent on the two-signal model of activation [58]. The first signal is triggered by the T cell receptor (TCR) that recognizes the cognate antigen peptide presented by the major histocompatibility complex (MHC) on the surface of professional antigen-presenting cells (APCs), such as B cells, dendritic cells, and macrophages. That interaction results in the immunological synapse formation [59]. The TCR triggering alone is insufficient for an effective T cell response and leads to the effector cell anergy. For efficient T cell activation, the second signal from co-stimulatory receptors, such as 4-1BB or CD28, is indispensable [60,61], along with the cytokines produced by APCs that prime the T cell’ differentiation into a variety of effector types (a so-called third signal) [62].

4-1BB (CD137) is a co-stimulatory tumor necrosis factor receptor (TNFR) superfamily protein existing in both, monomeric and dimeric, forms [63]. 4-1BB is upregulated on activated CD4+ and CD8+ T cells and binds to its ligand, 4-1BBL (CD137L), expressed on APCs, including activated B-cells [64,65]. The interaction between 4-1BB and 4-1BBL recruits the TNF receptor-associated factor (TRAF) proteins, such as TRAF1, TRAF2 and TRAF3. That leads to a downstream signaling activation of NF-kB, PI3K/AKT, p38 MAPK, ERK1/2 and stress-activated protein kinase/JNK pathways [[66], [67], [68]]. As a result, 4-1BB signaling drives cytokine production, enhances anti-apoptotic protein production, mitochondrial metabolism and biogenesis, and promotes proliferation, memory formation and T cell survival [63,67,69].

CD28 is a homodimeric receptor belonging to the immunoglobulin superfamily of transmembrane proteins. CD28 is constitutively expressed on the surface of resting and activated T cells, both CD4+ and CD8+ [70]. The activation of CD28 is triggered by the extracellular domain binding to the ligands, including the B7 family members, CD80, CD86 or CD275, expressed on APCs [[71], [72], [73]]. The intracellular CD28 tail might be recognized by PI3Ks, GRB2, GADS, Fyn or Lck [[74], [75], [76], [77], [78]]. These interactions lead to the recruitment of NF-kB, NFAT and AP1 transcription factors further enhancing the cytokine, chemokine and anti-apoptotic proteins production, promoting proliferation, differentiation, survival and effector functions that were elegantly and broadly reviewed by others [79,80].

### 4.2 Functional characteristic of 4-1BB- and CD28-based CD19 CAR-T cells in preclinical studies

**Fig. 3:**
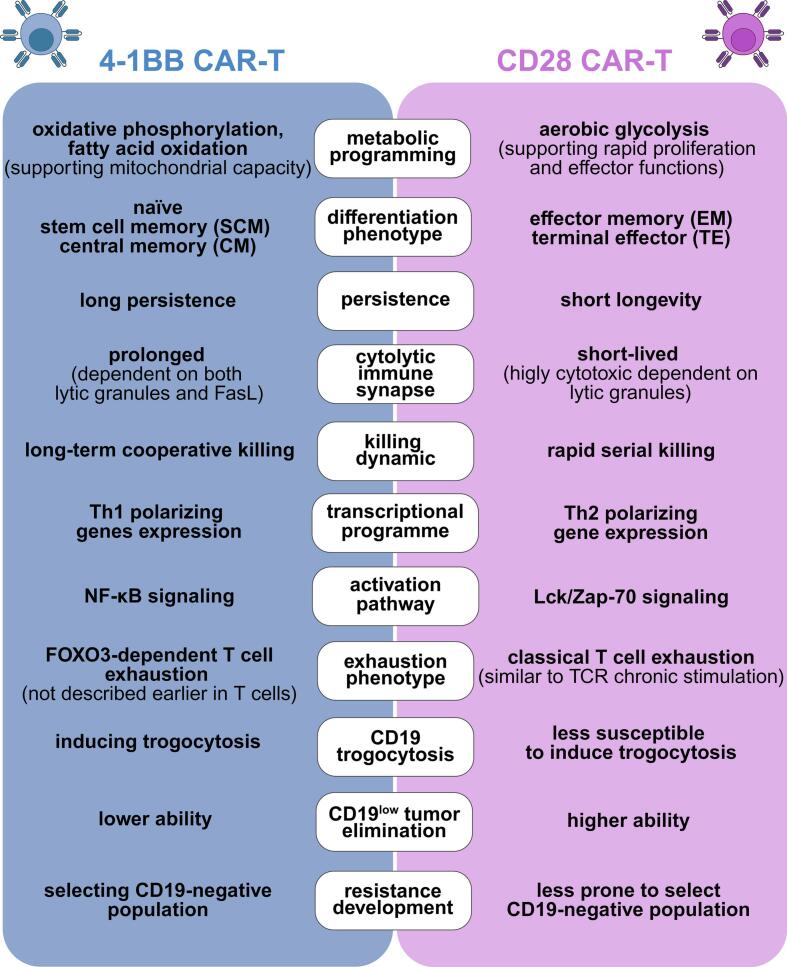
Functional properties of 4-1BB and CD28 CD19 CAR-T cells. CD19 CAR-T cells incorporating 4-1BB or CD28 costimulatory domain show distinct functional characteristics. 4-1BB CAR-T cells' advantages depend on their metabolic fitness promoting central memory differentiation, longer persistence and less exhausted phenotype while CD28 CAR-T cells superiority is demonstrated by their rapid serial killing, higher potential to eliminate CD19^low^ tumors, lower tendency to induce trogocytosis and to induce resistance.

The above-mentioned functions of the 4-1BB and CD28 co-stimulatory domains make them the ideal molecules for incorporation into the structure of CD19-targeted CARs to ensure the efficacy and survival of CAR-T cells. Many preclinical reports also show that 4-1BB- and CD28-based CD19-directed CAR-T cells exhibit distinct biological properties that shape their behavior, response dynamics, and long-term efficacy (Fig. 3).

These two types of CAR structures are distinct in terms of metabolic programming, T cell memory formation and differentiation phenotype. Importantly, accumulating evidence has identified CAR-T phenotypes that are correlated with durable responses to therapy, including a higher proportion of naïve and/or central memory subtypes [81,82], lower frequency of regulatory T cells (Tregs) [83,84] and T cells expressing exhaustion markers [85], and higher overall proliferative capacity [86].

The presence of 4-1BB in the CAR architecture preferentially drives oxidative phosphorylation and fatty acid oxidation, promoting mitochondrial biogenesis and the development of central memory CD8+ T cells with enhanced survival and proliferation potential [11,84,87,88]. Conversely, CARs utilizing the CD28 domain favor aerobic glycolysis, generating effector memory cells with rapid tumor-killing capabilities but reduced longevity [11,87]. This metabolic diversity aligns with clinical observations of improved CAR-T persistence when integrating the 4-1BB costimulatory domain into the CD19-targeting CAR structure [[89], [90], [91], [92]].

The preclinical observations that 4-1BB-based CARs demonstrate superior long-term tumor control and longer persistence, while CD28-based CARs exhibit faster tumor clearance [12,13], could also be explained by their immune synapse formation manner. 4-1BB CAR-T cells establish prolonged synapses that mediate cooperative chronic Fas ligand/lytic granules–based killing and greater persistence [93]. Additionally, the recruitment of the THEMIS-SHP1 phosphatase complex by 4-1BB-containing CARs tempers excessive CAR-CD3ζ phosphorylation, modulating activation intensity and mitigating premature exhaustion [94]. In contrast, CD28 CAR-T cells rapidly mobilize the lytic granules and form short-lived yet highly cytotoxic immune synapses, optimizing CAR-T ability for fast serial killing [93]. The rapid and highly cytotoxic synapse formation by CD28-based CARs is connected with the Lck-mediated phosphorylation of the CD3ζ-chain that leads to increased antigen-dependent T cell activation [94]. Furthermore, the stimulation of 4-1BB- and CD28-based CAR-T cells leads to the activation of different transcriptional programs. Activated CAR-T cells incorporating the 4-1BB domain show Th1 polarizing genes expression, whereas stimulation of CD28-based CAR-T cells enhances Th2 early polarizing genes expression [88]. Moreover, 4-1BB CAR-T cells maintain a unique regulatory profile enriched for memory-associated genes (TCF7, IL7R, LEF1) and HLA class II molecules [88,95].

In addition, 4-1BB CAR-T cells exhibit sustained activation of the NF-κB pathway, which enhances their survival and expansion, and is associated with anti-apoptotic proteins expression [96,97]. These effects are mediated by TRAF1, TRAF2 and TRAF3, which regulate NF-κB-dependent survival pathways [97]. On the other hand, the canonical and noncanonical NF-κB-dependent signaling is also responsible for the basal signaling observed in 4-1BB CAR-T cells, which can drive CAR-T cell aggregation and mediate its death by both Fas-dependent apoptosis and necroptosis [96,98,99]. Mechanistic studies reveal that 4-1BB sequesters A20 at the cell membrane in a TRAF-dependent manner, leading to NF-κB hyperactivation, ICAM-1-dependent cells' aggregation and necroptotic cell death [99]. While basal signaling is also observed through the Zap-70 pathway in CD28-based CAR-T cells [96], they are less prone to cell death by apoptosis and necroptosis than 4-1BB-based CAR-T cells [99]. Both 4-1BB- and CD28-based CARs experience dysfunction under chronic stimulation; however, they engage distinct phenotypes. Chronic 4-1BB activation induces FOXO3 transcriptional factors, initiating a state of T cell dysfunction not described earlier in T cells [95]. Contrastingly, CD28 CAR-T cells develop classical T cell exhaustion dependent on upregulation of genes such as PDCD1, LAG3, CTLA4, and NR4A1, similarly to chronic TCR stimulation [88,95].

Furthermore, 4-1BB- and CD28-based CAR-T cells differ in the dynamics of the tumor cells' killing that influences their ability to eradicate CD19^low^ antigen tumor cells and the susceptibility to trogocytosis-mediated antigen downregulation. Accordingly, CD19 CAR-T cells incorporating the CD28 domain surpass those with the 4-1BB domain in eliminating neoplastic cells with low expression of the CD19 antigen [100] and also outperform the 4-1BB-based ones by being less prone to CD19 trogocytosis [101]. Moreover, our recent study shows that long-term exposure of tumor cells to CD19-4-1BB CAR-T cells induces complete resistance accompanied by *CD19* genetic aberrations, including frameshift/missense mutations, as well as intron retention. In turn, prolonged exposure to CD28-based CAR-T cells decreases susceptibility to CD19 CAR-T therapy with only partial downregulation of CD19 antigen, not related to genetic changes [102]. Notably, by performing mathematical simulations, we showed that the probability of low-antigen tumor cells killing is the key factor contributing to antigen escape and resistance development. Specifically, we demonstrated that 4-1BB CAR-T cells are more prone than CD28 CAR-T cells to CD19-loss-related resistance development caused by genetic aberrations in the *CD19* gene. In contrast, CD28 CAR-T therapy induces CD19 downregulation (not complete loss), likely driven by epigenetic or other regulatory mechanisms different from those triggered by CD19-4-1BB CAR-T cells [102].

In summary, while CD19-4-1BB CAR-T cells are generally favored for their unique ability to persist in B-ALL patients [16,103], engineering a long-term persistent CAR-T cell that can recognize antigen-low targets would be a significant advance in the field. It has already been demonstrated that minor structural modifications, such as inserting additional ITAMs (immunoreceptor tyrosine-based activation motifs) into the zeta chain in the CAR, can adjust the antigen recognition threshold and enhance the capacity of 4-1BB-CARs to detect low-antigen targets while retaining superior persistence [100]. Notably, beyond differences in the costimulatory domain, the CAR constructs in tisa-cel and axi-cel also differ in their hinge-transmembrane regions. Intriguingly, a new CAR incorporating the CD28 H/T region along with 4–1BB and CD3ζ endodomains (CD19-CD28H/T-4–1BBζ) showed improved activity against CD19^low^ tumor cells, while preserving increased T cell persistence associated with 4-1BB [100]. This suggests it might serve as a superior backbone for further clinical development. Altogether, these findings pave the way for more effective utilization of costimulatory domains and precise engineering of CAR-T cell receptors for optimal recognition of target antigens on cancer cells while minimizing reactivity toward the same antigens expressed at lower levels on non-malignant tissues.

## 5 Resistance to CD19 CAR-T therapy

One of the most important limitations of CD19 CAR-T cells is the relapse, which affects about 30–40 % of treated patients [6,54]. The CD19-directed therapy failure could be a consequence of the manufacturing of CAR-T products and the CAR-T cells' quality, which have been broadly reviewed by others [104]. On the other hand, tumor cells can effectively evade immune surveillance and develop resistance mechanisms, among which the loss of the target antigen CD19 is the most widely studied [105].

### 5.1 Resistance mechanisms related to genetic aberrations of the *CD19* gene

**Fig. 4:**
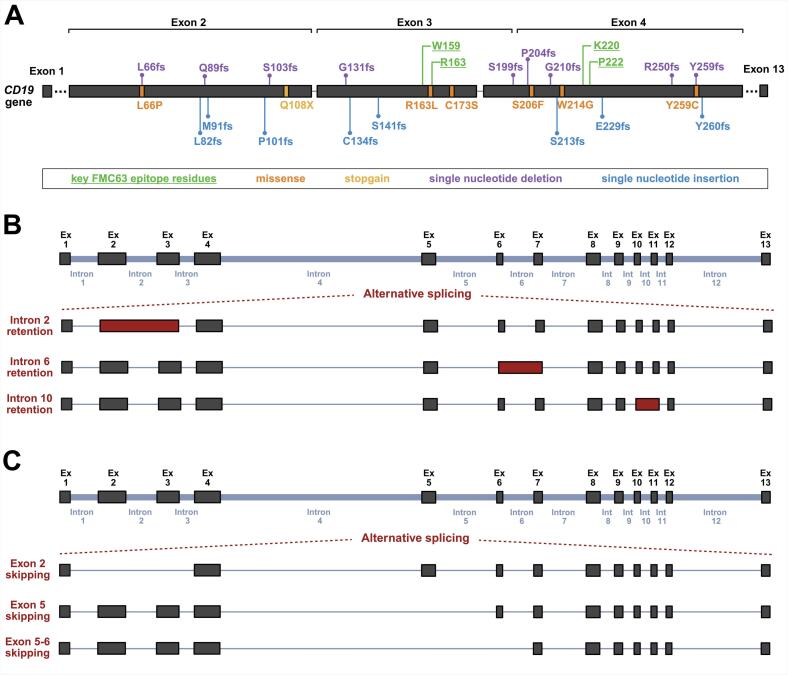
Genetic aberrations of the CD19 gene following FMC63-directed CAR-T therapy. The alterations of the CD19 gene after CAR-T cell administration caused by mutations with high risk of disruption of the FMC63 epitope (A) and alternative splicing events, including intron retention (B) or exon skipping (C).

CD19 antigen loss may result from epigenetic factors [10], hyperglycosylation [100], a lineage switch [101], or epitope masking [106,107]. Nevertheless, the most commonly reported cases are related to genetic aberrations of the *CD19* gene caused by mutations or alternative splicing (Fig. 4). These genetic changes lie at the bottom of inherent or acquired resistance to CD19 CAR-T therapy observed in patients [7].

In patients with B-ALL relapsing after treatment with tisagenlecleucel (tisa-cel), a variety of mutations in the *CD19* gene have been reported. These mutations affect exons 2-5 and include both frameshift and missense mutations, as well as large insertions or deletions, most of which result in premature termination of translation and loss of functional CD19 expression (Fig. 4A). Sotillo et al. (2015) first described truncating frameshift mutations in exon 2 (G67fs, G100fs) and exon 4 (P204fs), accompanied by loss of heterozygosity (LOH) or partial chromosome deletions affecting the *CD19* locus on chromosome 16. These events led to the production of aberrant *CD19* transcripts with disrupted FMC63 epitope targeted by CAR-T cells [9]. Subsequently, Orlando et al. (2018) expanded the spectrum of genetic changes in the *CD19* gene by identifying numerous aberrations across the paired (screening-relapse) samples from 12 B-ALL patients treated with tisa-cel [8]. These aberrations include: single-nucleotide variants (SNVs) in exon 2 (L66P), exon 3 (R163L) and exon 4 (W214G) resulting in non-synonymous CD19 coding; single nucleotide deletions in exon 2 (Q89fs, L66fs) and exon 4 (Y259fs), and single nucleotide insertions in exon 2 (L82fs, M91fs, P101fs), exon 3 (C134fs, S141fs, S213fs) and exon 4 (E229fs, Y260fs), both producing the frameshift and subsequently truncated CD19 protein; as well as larger deletions/insertions across exons 2–5, all resulting in premature stop codons and truncated protein. What is important, for every patient at least one aberration was detected after relapse, but not in samples before CAR-T administration [8]. More recently, Chen et al. (2023) confirmed recurrent truncating frameshift mutations in exon 2 (S103Lfs, Q108X – stopgain), exon 3 (G131Lfs), and exon 4 (S199Ffs, P204Lfs, G210Afs, R250Gfs) in B-ALL patients relapsed after tisa-cel treatment. Additionally, missense mutations (S206F, Y259C) and a nonframeshift deletion (W197del) were identified in exon 4, indicating diverse mutational mechanisms that may impair CD19 surface expression or alter the FMC63 epitope [108]. Although CD19-4-1BB CAR-T therapy is more commonly used in B-ALL treatment, there is a single report of a high-grade lymphoma patient who relapsed after a 4-1BB-based CAR-T product infusion with R163 mutation in the *CD19* gene [109].

In contrast to tisa-cel reports indicating multiple relapses with *CD19* mutation, the mutations after CD28-based axi-cel treatment are rarely reported and restricted to individual, mostly lymphoma, cases. Plaks et al. (2021) reported a missense mutation (C173S) in a single case of an axi-cel relapsed lymphoma patient, which was not present before treatment [110]. Another interesting case, reported by Ghobadi et al. (2022), was a subclonal deletion of codon Y260 in *CD19* in a B-ALL patient after blinatumomab treatment. This particular mutation was present following brexu-cel as the next line of treatment. Moreover, they also showed that deletion of this particular codon interfered directly with the binding of the FMC63-derived CAR scFv, representing a potent immune escape mutation [111]. A mutation in the same position was also reported earlier in B-ALL tisa-cel-treated patients as we mentioned above [8]. Recent comprehensive genomic analyses of 49 post-CAR-T therapy samples (45 axi-cel, 2 tisa-cel, 2 liso-cel) have demonstrated that only 5 of the analyzed cases harbor *CD19* aberrations. 4 out of these 5 patients had a durable CAR-T response and only 1 patient relapsed with clonal expansion of tumor cells with CD19 mutation, so the majority of studied patients retained expression of the wild-type CD19 protein [112]. These findings implicate non-antigenic resistance mechanisms after CD28-based CAR-T treatment of lymphoma patients, including i.e. APOBEC mutational signature [112], dysregulation of transcription factors such as PAX5, immune escape via CD274 (PD-L1) overexpression, and alterations in TMEM30A or IRF8 [113], as exemplary contributors to CAR-T resistance.

A better understanding of specific mutations in *CD19* and their role in the development of resistance to FMC63-targeted CAR-T therapy requires a deeper insight into the FMC63 binding site. The FMC63 epitope was mapped for the first time in 2019 [114]. Klesmith et al. described three amino acid residues (R163, P222 and W159) as fundamental for the FMC63 formation. They also showed that the R163 amino acid is a core of the FMC63 epitope, as every mutation of this residue results in epitope disruption. This was further confirmed in a study performed by He et al. [115], in which the K220 amino acid was additionally postulated as a residue important for FMC63 epitope recognition. Furthermore, the above-mentioned reports have shown that the FMC63 epitope is formed by the residues encoded in exons 3 and 4 of the *CD19* gene; therefore, any mutation causing protein truncation in this region or upstream disrupts recognition by FMC63-targeted CAR-T cells. Moreover, our recent study in the B-ALL cell line exposed to CD19-4-1BB CAR-T cells in vitro indicated that the R163 mutation is directly associated with resistance to CD19 CAR-T cells. We observed that this particular mutation produced a specific loss of the FMC63 epitope and affected the recognition by CAR-T cells [102], being in accordance with observations from clinical reports, where the R163 mutation was detected in patients relapsing after CD19-directed CAR-T therapy [8,109].

CD19 aberrations may also arise from alternative splicing of the transcript of the *CD19* gene, such as intron retention and exon skipping (Fig. 4B, C).

Intron retention in transcript results in a nonfunctional *CD19* isoform that can contribute to the antigen loss and failure of CD19-directed therapy (Fig. 4B) [116]. The retention of intron 2 in the CD19 transcript was reported in B-ALL patients who relapsed after 4-1BB-based tisa-cel treatment [8,117,118]. Moreover, the CD19 isoform with intron 2 retention was also detected in CD19-negative subclones in B-ALL samples before CAR-T therapy, raising the risk of therapy failure [116]. The isoforms of CD19 retaining intron 2, 6 or 10 were also observed in lymphoma patients after CD19 CAR-T therapy [119], including CD28-based axi-cel CAR product [110].

Although the exon skipping does not disrupt the CD19 protein, it may contribute to CD19 loss, thus affecting the efficacy of CAR-T cells' response (Fig. 4C).

Accordingly, the alternative splice variants lacking exon 2 or exons 5-6 were detected in samples from relapsed B-ALL patients after tisa-cel therapy [9] and lymphoma patients after axi-cel treatment [110]. CD19 splice variants lacking exon 2 and/or exons 5-6 were also detected in pediatric B-ALL patients and lymphoma patients at diagnosis and were postulated to cause primary resistance [110,120]. Additionally, it was demonstrated that CD19 isoform lacking exon 2 cannot bind to the tetraspanin CD81, which affects the proper transport of CD19 to the membrane, resulting in the accumulation of the misfolded CD19 protein in the endoplasmic reticulum and its downregulation on the cell surface [121].

Altogether, based on clinical reports *CD19* mutations contribute to FMC63 epitope loss and resistance in patients treated with CD19 CAR-T therapy. Interestingly, available results from clinical studies suggest that CD19 mutations are more frequently observed with CD19-4-1BB CARs (Table 2). In light of our experiments, the mechanistic explanation of this phenomenon is related to the attenuated response of CD19-4-1BB CARs to low antigen density tumors. As also discussed by others, it could be a result of sustained immune pressure exerted by long-persisting CD19-4-1BB CARs [100,122]. In contrast, the CD28-based post-therapy reports show that *CD19* mutations are relatively rare. The shorter persistence and a shortened period of immune pressure of the CD19-CD28 CAR-T cells may partially explain the low rate of CD19-negative relapses. Moreover, our in vitro models clearly show that CD19-CD28 CAR-T cells, by eliminating low antigen tumor cells, diminish the risk of tumor antigen escape. Furthermore, genetic aberrations of the *CD19* gene highlight the importance of the proper detection of the FMC63 epitope in clinical cases, both at diagnosis and relapse. As it was shown by our studies with in vitro resistance models [102] and in some clinical cases [111], only detection of FMC63 epitope provides reliable information on the target antigen levels available for FMC63-based CD19 CAR-T products. Conversely, the loss of the FMC63 epitope does not necessarily translate into the loss of the functional protein, so other CD19 epitopes could be targeted by alternative CD19 CAR-T products as the next-line treatment.

**Table 2:** Summary of the CD19 mutations studies in B-ALL and lymphoma patients after CD19-directed CAR-T therapy.

Study	Clinical Indication	CD19 CAR-T product (costimulatory domain)	Number of patients studied for *CD19* mutations	Number of patients harboring *CD19* mutations at relapse (%)	Ref.
Sotillo 2015	B-ALL	CTL019 (4-1BB)	4	3 (75 %)	[9]
Orlando 2018	B-ALL	CTL019 (4-1BB)	12	12 (100 %)	[8]
Chen 2023	B-ALL	CTL019 (4-1BB)	13	7 (54 %)	[108]
Zhang 2020	lymphoma	BinD19 (TCR-zeta/4-1BB)	1	1 (100 %)	[109]
Plaks 2021	lymphoma	Axi-cel (CD28)	6	1 (17 %)	[110]
Ghobadi 2022	B-ALL	KTE-X19, Brexu-cel (CD28)	1	1 (100 %)	[111]
Jain 2022	LBCL	Axi-cel (CD28) Tisa-cel (4-1BB) Liso-cel (4-1BB)	45 2 2	1 (2 %)	[112]

### 5.2 Other CD19 epitopes as alternatives for FMC63-loss relapses

Several CD19 epitopes, besides FMC63, are currently being evaluated in clinical trials for the treatment of various r/r B-cell malignancies. One of them is a low-affinity CAT scFv targeted by obe-cel, which was already approved for the treatment of r/r adult B-ALL, as we mentioned in chapter 2.1. The other epitopes that are still under evaluation are described below (Table 3).

**Table 3:** Alternative targets for anti-CD19 CAR-T therapy in clinical trials.

Institution (Country)	Clinical Indications	Targeted CD19 epitope	Hinge/TM/Costimulatory Domain/Signaling Domain	Clinical Trial's Number
MSKCC (USA)	r/r CLL or indolent B-cell lymphoma	SJ25C1	CD28/CD28/CD28/CD3ζ	NCT00466531
	r/r B-ALL			NCT01044069
	r/r aggressive B-NHL			NCT01840566
UCL (England)	relapsed CD19+ malignancy following allogeneic transplantation	4G7	CD8α/CD8α/4-1BB/CD3ζ	NCT02893189
IHBDH (China)	r/r B-ALL	HI19α	CD8α/CD8α/4-1BB/CD3ζ	NCT02975687
IDIBAPS (Spain)	r/r CD19+ leukemia or lymphoma	A3B1	CD8α/CD8α/4-1BB/CD3ζ	NCT03144583

MSKCC – Memorial Sloan Kettering Cancer Center; UCL - University College London; IHBDH - Institute of Hematology & Blood Diseases Hospital; IDIBAPS - Instituto de Investigaciones Biomédicas August Pi i Sunyer.

The first clinical trials with SJ25C1-targeting CD28 CAR-T cells showed limited efficacy in r/r CLL patients [89,123], whereas further clinical tests in r/r B-ALL revealed an 83 % CR rate with a 6.1 months median EFS [46]. Unfortunately, the MSKCC's CD19 CAR-T product also demonstrated relatively high toxicity, especially when administered following autologous stem cell transplantation in poor-risk r/r B-cell non-Hodgkin lymphoma (B-NHL) patients [124]. On the other hand, the epitope mapping studies indicated that the SJ25C1 epitope has a partially overlapping, yet not identical structure to FMC63, which does not exclude its utility in the treatment of some cases of relapse after FMC63-targeted therapies [115].

4G7, another epitope, incorporated into an allogeneic matched-donor CD19 CAR-T product, was evaluated in the CARD study in the treatment of B-ALL patients who had relapsed following allogeneic transplantation. This product revealed a tolerable safety profile and high efficacy with 83 % OS and 57 % EFS rates [125]. Moreover, although FMC63 and 4G7 epitopes are both centered around R163, they do not fully overlap and have different mutational tolerance of particular residues, including R163 [114]. This fact makes 4G7 epitope potentially useful in FMC63-negative relapses.

Another epitope of particular interest is HI19α targeted by inaticabtagene autoleucel (inati-cel), which was evaluated in r/r B-ALL patients in the NCT02975687 trial. Inati-cel demonstrated promising efficacy in a phase II study, achieving an ORR of 70.8 % at three months post-infusion, with durable responses observed during long-term follow-up. These findings, along with its manageable safety profile, led to the approval of inati-cel in November 2023 as the first CAR T-cell therapy for adult patients with r/r B-ALL in China [126]. Since inati-cel binds a different CD19 epitope than classical CAR T-cell therapies, it is a promising alternative for patients who experience relapse following FMC63-directed treatments [127].

A3B1 is an additional example of a promising epitope currently under investigation. It is targeted by varnimcabtagene autoleucel (var-cel), evaluated in the NCT03144583 trial for r/r B-ALL and B-NHL treatment. In this study, var-cel demonstrated 68.6 % OS and 47 % PFS at 1-year in B-ALL patients. In B-NHL patients the ORR at day 100 was 75 % and CRR was 50 % [128]. Although this product has not yet been approved for r/r B-NHL and is currently in the stage of updating long-term follow-up results, it has been granted a Hospital Exemption status in Spain for adult patients with r/r B-ALL and has received PRIority MEdicines (PRIME) designation from the EMA as the first academic CAR-T-cell therapy [129].

### 5.3 The role of other signaling regions in CAR efficacy

Costimulatory domains are not the only domains determining the efficacy of CAR-T cell therapy. While costimulatory domains incorporated into CAR constructs deliver an activating signal upon CAR recognition of the tumor antigen, it has been recently demonstrated that hinge and TM regions are also involved in CAR activation. They directly modulate this process through interactions with endogenous costimulatory molecules, which must undergo di−/trimerization at the cell surface to recruit the appropriate signaling molecules and to mediate T cell activation [130,131]. The role of hinge and TM regions was demonstrated using constructs comprising the FMC63 scFv and a 4-1BB costimulatory domain combined with either CD8α, CD28, or IgG4 hinge, as well as CD8α or CD28 TM. CARs containing CD28 hinge and CD28 TM facilitated the most robust recruitment of endogenous CD28, which amplified the activation signal and sensitized the CAR-T cells to lower antigen levels. When hinge and TM were derived from the CD8α molecule, heterodimerization with endogenous CD28 was also observed, albeit to a lesser extent. In contrast, less efficient dimerization with endogenous costimulatory molecules was detected with the IgG4 hinge. This can be explained by the presence of an unpaired cysteine residue, which occurs in both the endogenous CD28 and the CD28/CD8α hinge/TM but is absent in the IgG4 hinge [132]. Reports on cysteine-mediated clustering and its impact on CAR-T cells' efficiency have been further corroborated by subsequent research [133].

The superiority of the CD28 hinge/TM in FMC63-based CARs was also confirmed by increased recruitment of the ZAP70 protein to the intracellular portion of the CAR, as demonstrated by live imaging of tumor cell killing by single CAR-T cell. Moreover, CARs incorporating a CD28 hinge/TM and a 4-1BB costimulatory domain exhibited comparable activity to CARs containing a CD28 hinge/TM and a CD28 costimulatory domain in in vivo experiments against CD19^low^ leukemia [100].

Furthermore, modifications of the intracellular activation domain have also been shown to affect the anti-tumor activity of CAR-T cells. In all currently FDA-approved anti-CD19 CAR-T therapies, this domain corresponds to the CD3ζ chain, which contains three ITAMs. Comparative experiments have been conducted with CARs incorporating double CD3ζ (CD3ζζ), standard CD3ζ, and truncated CD3ζ containing a single ITAM (CD3ζ**). Results indicate that elimination of low-antigen cells by CAR-T cells increases proportionally with the number of ITAMs, with the CD3ζ** CAR exhibiting the lowest activity and the CD3ζζ CAR the highest [100].

### 5.4 New concepts in CAR design

While classic second-generation CARs achieved remarkable clinical success, ongoing research has focused on refining these constructs to overcome remaining limitations, improve tumor eradication and decrease relapse rates. Accordingly, the development of third-generation CARs incorporating an additional costimulatory domain was aimed to produce a synergistic effect and further enhance anti-tumor activity. However, in vivo studies revealed that, despite improved persistence and proliferation of third-generation CAR-T cells, no superior eradication of hematologic malignancies was achieved compared with second-generation CAR-T cells, as reviewed by others [134]. The other reported limitations include more rapid exhaustion and more frequent induction of immune-related adverse events in the case of third-generation CAR-T cells than their second-generation counterparts [135].

Among innovative strategies designed specifically to prevent the development of resistance to CAR-T therapy are “OR” logic gates, which are designed to enable CARs to recognize two distinct antigens, thereby diminishing the chances of tumor antigen escape [134,136]. Such designs include DuoCAR and Tandem CAR constructs. The DuoCAR consists of two independent CARs co-expressed on the surface of the same T cell. In leukemia models, DuoCAR-T cells targeting both CD19 and CD22 were more effective in eradicating tumor cells than the sequential administration of CAR-T cells targeting one of these antigens, individually. Contrastingly, Tandem CARs represent a single CAR molecule equipped with two scFvs. Early-phase clinical trials have demonstrated encouraging efficacy of Tandem CAR-T cells targeting CD19/CD20 and CD19/CD22 in the treatment of B-cell malignancies, which was elegantly reviewed by others [136]. It should be emphasized, however, that increasing the number of target antigens, as occurs with “OR” logic gates, may also increase the risk of on-target, off-tumor effects. This underscores the need for rigorous safety evaluations of therapies based on such approaches [134,136]. One way to control the activity of CAR-T cells and ensure their safety is to equip them with synthetic receptors such as killing, adapter, small-molecule switches or Boolean logic gating (AND, OR, and NOT gates). These engineered molecules, when incorporated simultaneously with the CAR structure, could activate or inhibit the function of CAR-T cells under specific conditions thereby enhancing their safety, increasing specificity against tumor cells, improving persistence or reducing exhaustion. Different examples of switch receptors were already broadly reviewed by others [[137], [138], [139], [140]].

Another crucial aspect of improving CAR design is enhancing the efficacy of CAR-T cells against low-antigen tumors. Evidence suggests that several specific modifications can enhance the functional features of CAR-T cells, enabling them to more effectively target low-antigen tumors (Table 4). One such modification includes adding the ITAM domain by multiplying the CD3ζ chain or altering the hinge/transmembrane domains (H/TM) from CD8α to CD28 in a classical 4-1BB-based CAR structure, as it was demonstrated for CAR-T cells targeting CD19, HER2 and GPC2 [100,141]. Another significant improvement is the incorporation of the intracellular domain of LAT (linker for activation of T cells) into the CD19 CAR and the development of a CD22xCD19-LAT bicistronic CAR. This construct exhibited a stronger effect against low-antigen leukemia cells compared to the conventional 4-1BB-based CD22 CAR [142]. Furthermore, enhancing CAR-T cells with the canonical AP-1 factor c-Jun or the cytosolic signaling adaptor molecule SLP-76 (MT-SLP-76) has also shown to improve their ability to eliminate low-antigen tumors [143,144].

**Table 4:** CAR design optimization to improve functional features and effectively eradicate low-antigen-density tumors.

CAR design optimization	CAR domains structure	CAR target	Efficacy against low-antigen tumors	Ref.
Additional ITAM domains	CD8αH/TM-4-1BB-CD3ζζ	CD19	Higher efficacy than classical CAR design	[100]
Mixed hinges-TM domain	CD28H/TM-4-1BB-CD3ζ	CD19 HER2 GPC2	Higher efficacy than classical CAR design	[100,141]
LAT	CD8αH/TM-4-1BB-CD3ζ CD28H/TM-LAT	Bicistronic: CD22xCD19-LAT	Higher efficacy of bicistronic CAR than CAR-CD22 alone	[142]
c-Jun	CD28H/TM-CD28-CD3ζ CD8αH/TM-4-1BB-CD3ζ	CD19 GD2	Higher efficacy, increased cytokine production and resistance to exhaustion	[143]
SPL-76	CD28H/TM-4-1BB-CD3ζ CD8αH/TM-4-1BB-CD3ζ IgG4H-CD28TM-4-1BB-CD3ζ	CD19 CD22 ROR1 BCMA HER2	Higher efficacy than no SPL-76 counterparts	[144]

Based on the previous reports, the most frequently utilized CAR structure for optimization primarily relies on 4-1BB costimulation variants (Table 4). This particular CAR version is likely preferred due to its longer persistence. However, while enhancing CD19-4-1BB CAR constructs to more effectively target low-antigen tumors could help mitigate the issue of antigen escape, it is worth to underline that the extended persistence of 4-1BB-based CARs can also be a double-edged sword, potentially contributing to the development of resistance.

## 6 Conclusions

The design of CAR-T cells has evolved significantly over the past decades, driven by the need to improve efficacy, persistence, and safety of the therapy. In this context, the costimulatory domain has emerged as one of the critical factors shaping the response of CD19-directed CAR-T cell therapies. The two main signaling platforms used in clinical products, 4-1BB and CD28, induce distinct metabolic, transcriptional, and functional programs that profoundly shape CAR-T cell phenotype, persistence, and mechanisms of tumor clearance. While 4-1BB-based CAR-T cells promote long-term persistence through enhanced mitochondrial metabolism, memory formation, and sustained NF-κB signaling, they are also more susceptible to activation-induced cell death and immune escape mechanisms, including antigen loss and trogocytosis. In contrast, CD28-based CAR-T cells exhibit rapid effector functions and superior ability to eliminate CD19^low^ tumor cells, but at the cost of shorter persistence and a higher tendency toward classical exhaustion.

These biological differences are reflected in clinical outcomes. The long-term follow-up clinical studies and real-world comparative analyses consistently indicate that CD19-CD28 CAR-T cell therapies demonstrate superior clinical efficacy compared to CD19-4-1BB products in lymphoma patients, achieving higher response rates, longer progression-free and overall survival, and more durable remissions – albeit with increased toxicity. Comprehensive meta-analyses, including the large-scale review performed by Montagna et al., further support that CD28 incorporation into CAR architecture enhances treatment outcomes, reinforcing its clinical advantage over 4-1BB-based constructs. In B-ALL patients, however, both CAR constructs show comparable efficacy, though differences in relapse patterns and toxicity profiles persist.

The functional differences between both costimulatory domains may also influence the mode and likelihood of resistance development, with 4-1BB CARs more readily driving CD19-negative relapse. This is supported by several reports on CD19 loss relapses in B-ALL patients treated with 4-1BB-based tisa-cel caused by genetic aberrations in the *CD19* gene. These alterations induce FMC63 epitope loss or CD19 protein disturbance by affecting exons 2-5. Specifically, mutations of R163, a central residue involved in FMC63 epitope formation, are recurrent and functionally validated. In contrast, for CD28 CAR-T therapies (mainly axi-cel), *CD19* mutations are rarely reported, with resistance driven by non-antigenic mechanisms such as transcriptional reprogramming.

Altogether, recent findings underscore the higher susceptibility of 4-1BB CAR-T cells to epitope loss-driven escape, emphasizing the need for monitoring the epitope level in patients at both diagnosis and relapse. Furthermore, they also support the clinical potential of alternative CD19-targeting CAR-T cells that recognize different epitopes, offering options for salvage therapy after FMC63-based CAR-T therapy failure.

Given this context, the CD19 CAR-T field may face a significant dilemma. While 4-1BB-based CAR-T products remain more widely used, their higher association with antigen loss and resistance may call into doubt their long-term efficacy in some hematological malignancies. This raises the question of whether future efforts should focus on improving CRS management, enhancing the persistence and reducing the toxicity of CD28-based CAR-T cells while maintaining their strong anti-tumor efficacy, or improving the 4-1BB-based CAR-T cells to increase their ability to eradicate low-antigen tumors. Importantly, while CAR construct design in promoting persistence and antitumor efficacy has been widely discussed, clinical evidence also suggests the CAR-T cell product's manufacturing process plays a critical role in the clinical impact and the toxicity profile experienced by patients after infusion [145]. Therefore, the manufacturing process as such is currently extensively optimized to harmonize the competing interests of enhancing CAR-T cell efficacy and minimizing toxicity. Accordingly, various process parameters in each key manufacturing step have been monitored to better characterize the impact of manufacturing variables that influence CAR-T cell product performance (reviewed in [146]). Moreover, a novel high-dimensional framework for profiling CAR-T cells throughout manufacturing has been recently established. By capturing key attributes driving CAR-T behavior and function, this platform will help to align product features, including various costimulatory domains, with specific therapeutic goals [147].

In summary, while CAR-T cells hold great promise, T cell functionality in clinical settings remains a critical challenge. Understanding this issue is essential for enhancing the efficacy and guiding the next generation of cell therapy design.

## Declaration of generative AI in scientific writing

OpenAI's ChatGPT tool was used exclusively to improve the readability and language of selected parts of the manuscript. All materials obtained using ChatGPT were then reviewed and edited by the authors.

## Funding statement

The work was supported by the Polish 10.13039/501100004281National Science Centre (grant no. 2023/49/N/NZ7/03096 to MK, 2023/50/A/NZ6/00423 to MW, 2020/39/O/NZ6/01434 to MW) and 10.13039/501100000781European Research Council (805038/STIMUNO/ERC-2018-STG to MW).

## Declaration of competing interest

The authors declare that they have no known competing financial interests or personal relationships that could have appeared to influence the work reported in this paper.

## Data Availability

No data was used for the research described in the article.
